# Inhibition of *Staphylococcus aureus* Adhesion to the Surface of a Reticular Heavyweight Polypropylene Mesh Soaked in a Combination of Chlorhexidine and Allicin: An *In vitro* Study

**DOI:** 10.1371/journal.pone.0126711

**Published:** 2015-05-11

**Authors:** Bárbara Pérez-Köhler, Francisca García-Moreno, Yves Bayon, Gemma Pascual, Juan Manuel Bellón

**Affiliations:** 1 Department of Surgery, Medical and Social Sciences. Faculty of Medicine and Health Sciences. University of Alcalá. Madrid, Spain; 2 Covidien—Sofradim Production, Trévoux, France; 3 Department of Medicine and Medical Specialties. Faculty of Medicine and Health Sciences. University of Alcalá. Madrid, Spain; 4 Networking Research Center on Bioengineering, Biomaterials and Nanomedicine (CIBER-BBN). Madrid, Spain; University Hospital of the Albert-Ludwigs-University Freiburg, GERMANY

## Abstract

**Introduction:**

Presoaking meshes for hernia repair with antiseptics prior to implantation could decrease the adhesion of microorganisms to the material surface and reduce the risk of antibiotic resistances. In this work, we evaluate chlorhexidine and allicin (natural antiseptic not yet tested for these purposes) against vancomycin as antiseptics to be used in the pretreatment of a heavyweight polypropylene mesh using an *in vitro* model of bacterial contamination.

**Methods:**

Solutions of saline, vancomycin (40 µg/mL), allicin (1,000 µg/mL), chlorhexidine (2%-0.05%) and the combination allicin-chlorhexidine (900 µg/mL-0.05%) were analyzed with agar diffusion tests in the presence of 10^6^ CFU *Staphylococcus aureus* ATCC25923. Additionally, sterile fragments of Surgipro (1 cm^2^) were soaked with the solutions and cultured onto contaminated agar plates for 24/48/72 h. The antimicrobial material DualMesh Plus was utilized as positive control. At every time, the inhibition zones were measured and the bacterial adhesion to the mesh surface quantified (sonication, scanning electron microscopy). Cytotoxicity of the treatments was examined (alamarBlue) using rabbit skin fibroblasts.

**Results:**

The largest zones of inhibition were created by allicin-chlorhexidine. Chlorhexidine was more effective than vancomycin, and allicin lost its effectiveness after 24 h. No bacteria adhered to the surface of the DualMesh Plus or the meshes soaked with vancomycin, chlorhexidine and allicin-chlorhexidine. On the contrary, saline and allicin allowed adherence of high loads of bacteria. Vancomycin had no toxic effects on fibroblasts, while allicin and chlorhexidine exerted high toxicity. Cytotoxicity was significantly reduced with the allicin-chlorhexidine combination.

**Conclusions:**

The use of antiseptics such as chlorhexidine, alone or combined with others like allicin, could represent an adequate prophylactic strategy to be used for hernia repair materials because soaking with these agents provides the mesh with similar antibacterial properties to those observed after soaking with vancomycin, similar to the effect of DualMesh Plus.

## Introduction

In general surgery, hernia repair is one of the most frequently performed procedures [1]. Over time, the surgical techniques designed for the treatment of these procedures have evolved from autoplasty, using tissues from the patient, to the implantation of prosthetic materials. These latter materials, together with the data provided by Lichtenstein's group [2], have shown the compulsory necessity of utilizing biomaterials to repair hernia defects. In such settings, infection of the surgical site or the prosthetic mesh is a clinical complication that has an important social impact and increased economic costs [3].

The biomaterials utilized most often in hernia repair are made of reticular polypropylene (PP). There are several variants of PP meshes available; however, their use, as with all biomaterials, is not exempt from post-surgical complications, with infection being one of the most devastating effects. The incidence of infection following a prosthetic inguinal hernia repair ranges from 3–4%, and this rate is even higher in the case of incisional hernia repair, with infection rates ranging from 6 to 10% of the total procedures [4]. These percentages are of great importance given the high frequency of the hernia repair procedures using biomaterials. Only in the USA does the prosthetic mesh infection affect approximately 30,000 patients per year and 3,000 patients per year in the cases of inguinal and incisional hernia repair [5]. The prevalence of mesh infection is higher in incisional repairs because, in most cases, the genesis of these hernias was fundamentally motivated by a bacterial contamination during the laparotomy prior to the development of the hernia. Moreover, some patients have been subjected to several surgical procedures due to recurrences; therefore, parts of the potentially contaminated material could stay attached to the patient’s tissues [6].

Preoperative antibiotic prophylaxis is one of the most commonly utilized strategies in hernia repair [7], although the published results regarding the effectiveness of this strategy are controversial [8,9]. One alternative to preventing implant infection could be achieved by avoiding colonization of the mesh and surrounding tissue by microorganisms during the early stages of contamination, thus inhibiting bacterial adhesion. This approach would also prevent subsequent biofilm formation onto the mesh surface, which is critical because the biofilm structure provides protection from antibiotics and enhances bacterial adhesion to the mesh surface [10,11]. By avoiding this, the tissue integration and vascularization of the implant would be favored, stimulating the formation of a protective tissue layer containing host immune cells, mainly macrophages, and competitively preventing the bacterial colonization of the implant [12].

Taking into account that the use of antibiotics can lead to the development of novel resistant bacterial strains, several non-antibiotic antibacterial agents are being used in combination with either surgical procedures or medical devices. In this regard, chlorhexidine (CHX) is utilized in the coating of vascular catheters [13] and in the topical lavage of the surgical site, which is important because the majority of pathogens responsible for causing material infections are present on the patient’s own skin [14]. Furthermore, CHX in combination with silver carbonate has been successfully used to coat a laminar expanded polytetrafluoroethylene (ePTFE) mesh material for hernia repair in the setting of an infection [15].

Another non-antibiotic antibacterial agent is allicin, a natural antiseptic compound derived from garlic cloves that was recently used in an experimental model of prosthetic joint infection, alone and in combination with vancomycin [16]. This antiseptic is known to exert a potent activity against both gram-positive and gram-negative bacteria [17]. Additionally, *in vitro* studies indicate that allicin inhibits the synthesis of some bacterial adhesins such as the polysaccharide intracellular adhesin (PIA) responsible for biofilm formation by *Staphylococcus epidermidis* (*Se*) [18,19].

Soaking the mesh with antiseptic antibacterial agents, such as allicin or CHX, constitutes a simple and economic strategy for the control of bacterial adhesion to the mesh surface at the moment of the implant. Currently, there are insufficient data regarding the potential use of mesh soaking for the prevention of hernia mesh infection. Accordingly, the aim of the present work was to test, under *in vitro* conditions, the performance of a reticular heavyweight PP mesh soaked with different antibiotic or antiseptic treatments and exposed to an environment previously contaminated with *Staphylococcus aureus*.

## Materials and Methods

### Antibacterial solutions to be tested

The following sterile antibacterial aqueous solutions were utilized:

- Saline: Sodium chloride (NaCl) 0.9% (negative control).
- Vancomycin: Vancomycin 40 μg/mL (Hospira, Madrid, Spain).
- Allicin: Allimed Liquid (Allicin International Ltd., Stratford, UK), containing 1000 μg/mL of stabilized allicin.
- Chlorhexidine digluconate (CHX): CHX 0.05% (Santa Cruz Biotechnology, Texas, USA).
- Allicin-CHX: Mixed solution containing allicin 900 μg/mL and CHX 0.05%.

### Preparation of the bacterial inocula

The bacterial strain *Staphylococcus aureus* ATCC25923 (*Sa*) was utilized. To perform the experiments, a cryovial containing *Sa* was thawed, plated on Lysogeny Broth (LB) agar plates and incubated for 24 h at 37°C. A single colony from each plate was inoculated into 25 mL of LB medium and kept overnight at 37°C. The absorbance (OD600) was read by spectrophotometry and adjusted with sterile 0.9% saline to an OD600 equivalent to approximately 1 x 10^8^ CFU/mL. Two tenfold serial dilutions were performed to generate a 10^6^ CFU/mL inoculum. The number of viable bacteria in every inoculum was determined with the spot plaque method, in triplicate.

### Titration of CHX

Prior to the evaluation of the antimicrobial solutions, a titration of CHX was developed with the aim of selecting the lowest dose exhibiting intense antibacterial activity. To perform this step, several CHX concentrations (2%, 1%, 0.5%, 0.2%, 0.1% and 0.05%) were prepared by dilution in sterile ultrapure water and subsequently tested using the agar well diffusion method. Using sterile swabs, bacterial lawns were spread onto 30 LB agar plates using 1 mL of the *Sa* 10^6^ CFU/mL inoculum. Circular wells (8 mm in diameter, 4 mm in depth) were punched in the middle of each plate and filled with 100 μL of the corresponding CHX dose (n = 5 per concentration). Plates were incubated at 37°C for 24 h, 48 h and 72 h. At each of the time points, the zones of inhibition were recorded by measuring two perpendicular diameters per plate. The results were expressed as the mean inhibition zone (mm) per CHX concentration.

### Antimicrobial activity test

The effectiveness of the different antibacterial solutions was assessed using the agar well diffusion method, with the same methodology utilized in the titration of CHX (n = 5 plates per solution). After 24 h, 48 h and 72 h of incubation at 37°C, the zones of inhibition were measured and the results expressed as the mean inhibition zone (mm) per study group.

### Soaking the mesh fragments

The antimicrobial solutions were utilized to soak fragments of a PP biomaterial for hernia repair. The monofilament Surgipro mesh (Covidien, Dublin, Ireland) was cut under sterile conditions into squares (1 cm^2^), dipped in 20 mL of the corresponding solution for 1 min (n = 21 fragments per study group) and placed individually onto pre-lawned LB agar plates. Sterile fragments of the antimicrobial mesh DualMesh Plus (DM+) (W. L. Gore & Associates Inc., Delaware, USA) were used as a positive control and processed under the same conditions. The plates were incubated at 37°C for 24 h, 48 h and 72 h. At each of the time points, the zones of inhibition were recorded as previously described, and the mesh fragments were collected to evaluate the bacterial adhesion to the mesh surface through sonication and scanning electron microscopy protocols.

### Sonication of the mesh fragments

At every time point, 5 samples from each study groups were collected. The mesh fragments were carefully washed in 1 mL of sterile 0.9% saline to remove the non-adherent bacteria and immediately immersed in sterile glass tubes containing 10 mL of peptone water. The tubes were sonicated for 10 min at 40 KHz using a Bransonic 3800-CPXH ultrasonic cleaning bath (Branson Ultrasonics, Connecticut, USA). The supernatant of each tube was thoroughly vortexed for 1 min, serially diluted in peptone water (7 tenfold dilutions), and the bacterial recovery in each sample was determined with the spot plaque method. The colonies on each plate were counted after 24 h of incubation at 37°C. The minimum level of detection was established at 3 x 10^2^ CFU/mL. The results were expressed as the mean number of viable bacteria per mesh fragment.

### Scanning electron microscopy

Two mesh fragments per study group per time point were collected, fixed in 3% glutaraldehyde for 2 h and washed in Millonig buffer (pH 7.3). The samples were then dehydrated in an increasing graded ethanol series (30%, 50%, 70%, 90%, 100%, incubation for 15 min each), brought to the critical point in a Polaron CPD7501 (Fisons Instruments, Ipswich, UK), gold-palladium coated, and visualized in a Zeiss DSM950 scanning electron microscope (Carl Zeiss, Oberkochen, Germany).

### Cell viability

To determine whether the antibacterial treatments affected the growth rate of cultured cells, a viability test was performed using the alamarBlue assay (AbD Serotec; Bio-Rad Laboratories Inc., California, USA), a colorimetric method to measure cell proliferation and cytotoxicity. This assay was conducted using fibroblasts isolated from the dermis skin of New Zealand White rabbits by the explant method, as described below.

Small biopsies (approximately 1 cm^2^) were collected at the Animal Research Center of Alcalá University. In order to accomplish with the animal welfare 3R’s criteria, the biopsies were obtained from 3 euthanized rabbits which had previously been utilized in another study, whose protocols were approved by the Committee on the Ethics of Animal Experiments of the University of Alcalá (registered code: ES280050001165). The animals were euthanized in a CO_2_ chamber. All the protocols belonging to that study were developed in strict accordance with the recommendations set forth in the Guide for the Care and Use of Laboratory Animals of the National and European Institutes of Health (Spanish law 32/2007, Spanish Royal Decree 1201/2005, European Directive 2010/63/UE and European Convention of the Council of Europe ETS123).

The biopsies were immersed in minimal essential medium (MEM) (Life Technologies Corporation, California, USA) and immediately processed under sterile conditions. The dermis was isolated with scalpel blades, diced into small explants and subsequently cultured in 25 cm^2^ flasks using low-glucose Dulbecco's modified Eagle medium (DMEM) supplemented with 10% fetal bovine serum (FBS), penicillin-G 10,000 U/mL, streptomycin 10,000 μg/mL and amphorericin-B 25 μg/mL (medium and reagents from Life Technologies Corporation). Culture flasks were kept in the incubator under a controlled humid atmosphere (37°C, 5% CO_2_) to allow the cells to migrate from the explant and colonize the flask surface. The media was changed every 3 days, and confluent cultures were treated with 0.25% trypsin-ethylenediaminetetraacetic acid (EDTA) at 37°C for 5 min, centrifuged at 200 g for 7 min, and transferred to culture flasks with 3 mL of complete DMEM, at a 1:4 ratio. The cells were visualized in a Zeiss Axiovert 40C phase-contrast microscope (Carl Zeiss).

Confluent third-passage cultures were trypsinized and centrifuged as previously described. The cells were then counted in a Neubauer hemocytometer, transferred to flat-bottom 96-well plates at a density of 1 x 10^4^ cells per well in complete DMEM and incubated overnight under a controlled atmosphere. Then, the media was discarded and replaced with 100 μL of fresh serum-free DMEM supplemented with different antibacterial treatments at the tested concentrations, in quadruplicate. Wells containing either 100 μL of serum-free medium and wells with treatments but no cells were utilized as blanks. Following 24 h of incubation at 37°C, 10 μL of the alamarBlue viability reagent were added to each well. Plates were incubated for 5 h at 37°C, and absorbance was measured at 570 nm and 600 nm using an iMark microplate absorbance reader (Bio-Rad Laboratories Inc.). Collected data were analyzed with the online alamarBlue colorimetric calculator provided by the manufacturer, available through the following link: http://www.abdserotec.com/colorimetric-calculator-fluorometric-alamarblue.html. The results were expressed as the mean percentage of alamarBlue reduction of treated cells versus non-treated cells. The assay was performed 3 times.

### Statistical analyses

The data collected from the inhibition halos, the sonication assays and the cell viability tests were compared between pairs of groups using the Mann-Whitney U test. The data were represented as the mean ± standard error of the mean (SEM), and tables containing the mean data, SEM and percentiles (minimum, 25%, median, 75%, maximum) were reported. All the statistical analyses were performed using the GraphPad Prism 5 computer package (La Jolla, California, USA) for Windows. The significance level was set at p<0.05.

## Results

### Quantification of the bacterial inocula

The bacterial suspensions used to perform all of the experiments contained a mean value of 1.48 x 10^6^ CFU/mL.

### Efficacy of the different CHX concentrations

**Fig 1:**
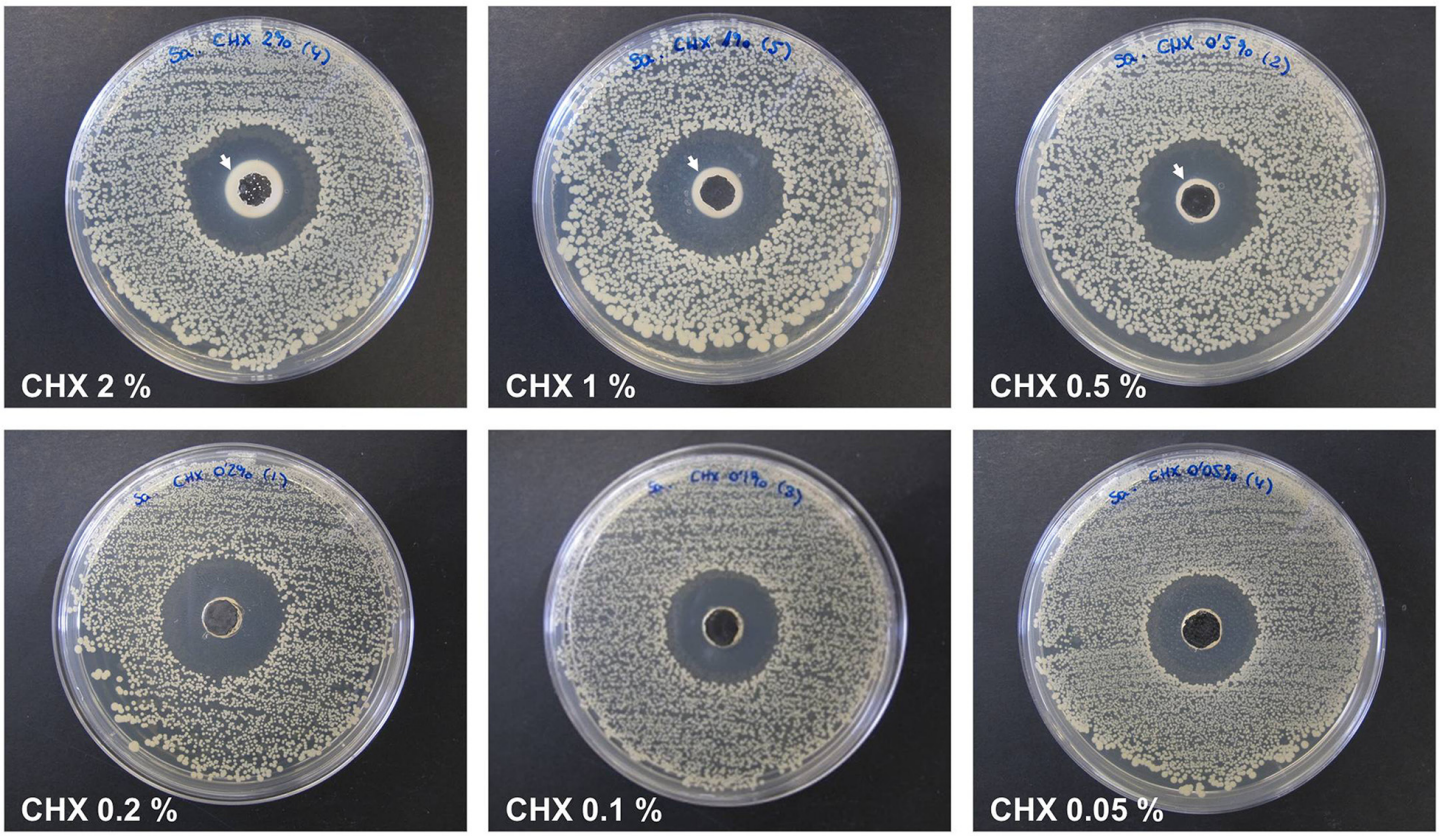
Titration of the different CHX concentrations. The images are representative for each concentration after 72 h of bacterial contamination. The plates from the highest CHX doses (2%, 1%, 0.5%) showed a dense precipitate (→) surrounding the wells punched in the agar.

All of the CHX solutions exhibited a strong antimicrobial activity against *Sa* at the 3 time points, regardless of the concentration tested. Despite this, the highest CHX doses (2%, 1% and 0.5%) produced a dense and opaque precipitate surrounding the wells punched in the agar (Fig 1) and were thus not considered to be adequate solutions. After 24 h, the mean diameter of the inhibition halos ranged between 27.40 ± 0.29 mm and 26.00 ± 0.27 mm (CHX 0.2% and 0.05%, respectively). Samples exhibited the same results at 48 h (27.70 ± 0.37 mm for CHX 0.2% and 25.60 ± 0.48 mm for CHX 0.05%) and 72 h (27.90 ± 0.37 mm for CHX 0.2% and 25.70 ± 0.34 mm for CHX 0.05%), At the 3 time points, the halos of the CHX 0.2% were statistically higher than those of the CHX 0.1% and CHX 0.05% (p<0.05), while the behavior of the CHX 0.1% and CHX 0.05% groups were very similar. Due to efficacy regarding the antibacterial activity of the lowest CHX concentrations tested, and considering the importance of selecting a low concentration to reduce the toxicity of the treatment, CHX 0.05% was chosen as the optimal dose to be utilized in the remainder of the assays. The Table 1 contains the mean data and percentiles of the 3 lowest CHX concentrations.

**Table 1:** Mean diameter, SEM and percentiles of the inhibition zones created by the 3 lowest CHX concentrations tested.

Mean diameter, SEM and percentiles of the inhibition halos (mm)				
CHX dose	Data	24 h	48 h	72 h
**CHX 0.2%**	Mean	27.40	27.70	27.90
	SEM	0.29	0.37	0.37
	Minimum	26.50	27.00	25.50
	25%	26.75	27.00	25.50
	Median	27.50	27.50	26.00
	75%	28.00	28.50	26.50
	Maximum	28.00	29.00	27.00
**CHX 0.1%**	Mean	26.50	26.80	26.60
	SEM	0.16	0.27	0.10
	Minimum	26.00	26.50	25.00
	25%	26.25	26.50	25.00
	Median	26.50	27.00	25.00
	75%	26.75	27.00	26.50
	Maximum	27.00	27.00	27.50
**CHX 0.05%**	Mean	26.00	25.60	25.70
	SEM	0.27	0.48	0.34
	Minimum	25.50	26.50	25.00
	25%	25.50	26.50	25.25
	Median	26.00	26.50	25.50
	75%	26.50	26.75	26.25
	Maximum	27.00	27.00	27.00

Data are collected from 5 samples per study group.

### Antimicrobial activity of the solutions

**Fig 2:**
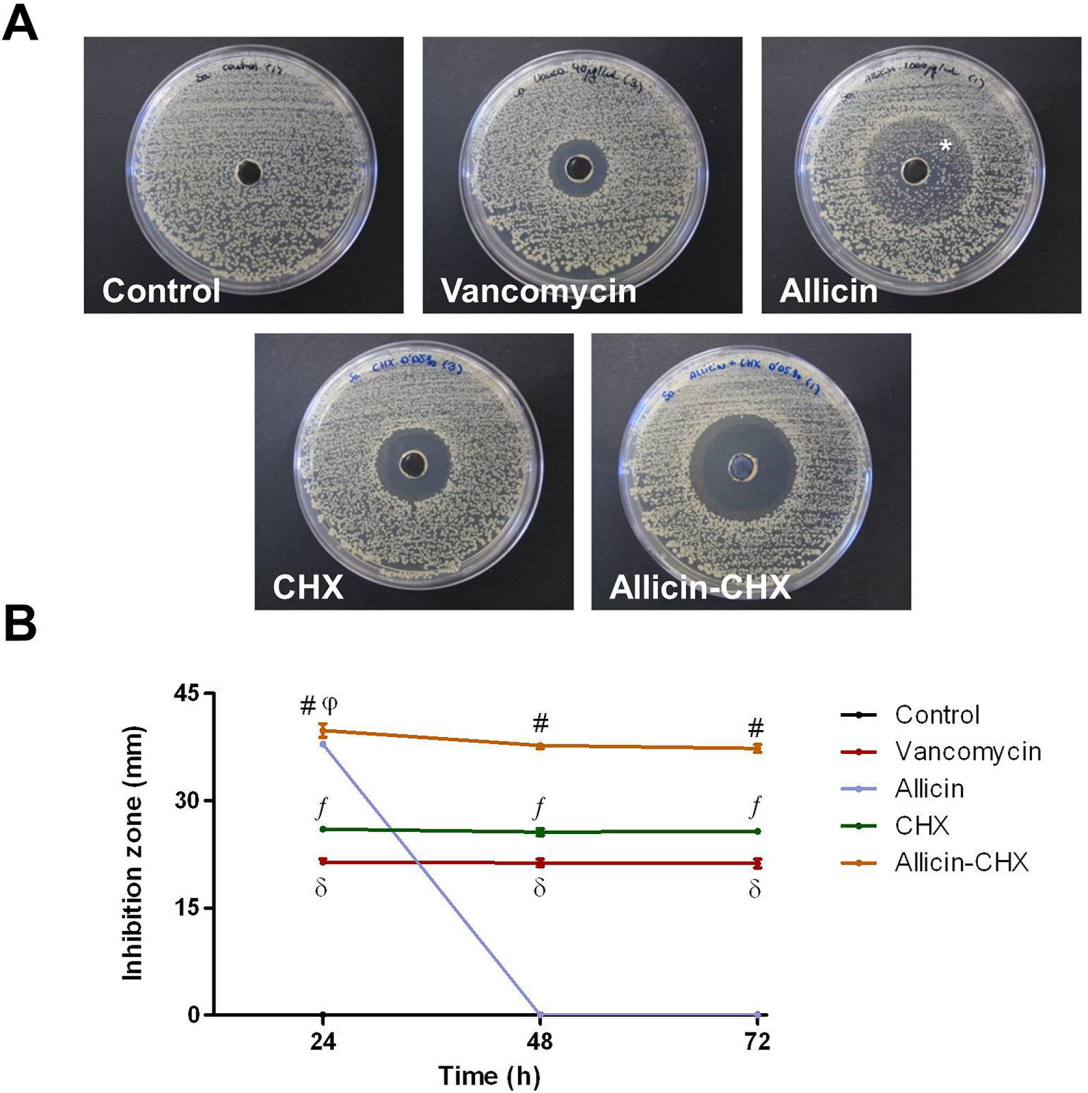
Zones of inhibition created by the different antibacterial treatments. (A) The images are representative for each study group at 72 h post-contamination with *Sa*. Note the intense bacterial growth in the control group and the bacterial colonization of the inhibition zones (*) in allicin-treated plates. (B) Mean diameter of the inhibition zones (mm) created by the antibacterial treatments over time. The results are expressed as the mean ± SEM for 5 samples inoculated with *Sa*. #: allicin-CHX vs control at 24/48/72 h (p<0.01); vs vancomycin, CHX at 24/48/72 h (p<0.05); vs allicin at 48/72 h (p<0.01). ƒ: CHX vs control at 24/48/72 h (p<0.01); vs vancomycin, allicin control at 24/48/72 h (p<0.05). δ: vancomycin vs control, allicin at 24/48/72 h (p<0.05). φ: allicin vs control at 24 h (p<0.01).

The antibacterial effect of the different solutions was evaluated with the well diffusion method after 24 h, 48 h and 72 h of contamination with *Sa* (Fig 2A). As expected, the control plates had no inhibition zone. With the exception of allicin, the behavior of the antibacterial solutions was consistent over time. The allicin-CHX treatment developed the widest inhibition zones at all of the time points analyzed. The behavior of the allicin solution was similar to allicin-CHX at 24 h, but surprisingly all the plates from the allicin group exhibited a bacterial colonization within the halos at 48 h onwards. Both CHX and vancomycin treatments behaved similarly, with the halos of CHX being slightly wider. At 24 h post-contamination, the control group showed statistical differences compared with the treated groups (p<0.01) due to the lack of inhibition halos in the agar plates (Fig 2B). At this time point, all of the antibacterial treatments were statistically different from one another (p<0.05) with the exception of the comparison between the allicin and allicin-CHX groups at 24 h, whose inhibition zones were almost identical. These results were very similar at 48 h and 72 h, but due to the bacterial colonization of the halos, allicin exhibited the same behavior as the control group both at 48 h and 72 h. The mean data, SEM and percentiles of the different groups and study times are reported in Table 2.

**Table 2:** Mean diameter, SEM, and percentiles of the inhibition zones created by the antibacterial solutions.

Mean diameter, SEM and percentiles of the inhibition halos (mm)				
Treatment	Data	24 h	48 h	72 h
**Saline**	Mean	No inhibition	No inhibition	No inhibition
	SEM	0	0	0
	Minimum	0	0	0
	25%	0	0	0
	Median	0	0	0
	75%	0	0	0
	Maximum	0	0	0
**Vancomycin**	Mean	21.40	21.30	21.20
	SEM	0.43	0.51	0.60
	Minimum	20.50	20.50	20.00
	25%	20.75	20.50	20.25
	Median	21.00	20.50	21.00
	75%	22.25	22.50	22.25
	Maximum	23.00	23.00	23.50
**Allicin**	Mean	37.90	Colonization	Colonization
	SEM	0.33	0	0
	Minimum	37.00	0	0
	25%	37.25	0	0
	Median	38.00	0	0
	75%	38.50	0	0
	Maximum	39.00	0	0
**CHX**	Mean	26.00	25.60	25.70
	SEM	0.27	0.48	0.34
	Minimum	25.50	25.00	25.00
	25%	25.50	25.00	25.25
	Median	26.00	25.00	25.50
	75%	26.50	26.50	26.25
	Maximum	27.00	27.50	27.00
**Allicin-CHX**	Mean	39.80	37.70	37.30
	SEM	0.93	0.44	0.51
	Minimum	38.00	36.50	36.00
	25%	38.00	36.75	36.25
	Median	39.50	38.00	37.50
	75%	41.75	38.50	38.25
	Maximum	43.00	39.00	39.00

Data are collected from 5 samples per study group.

### Effectiveness of the soaked meshes

**Fig 3:**
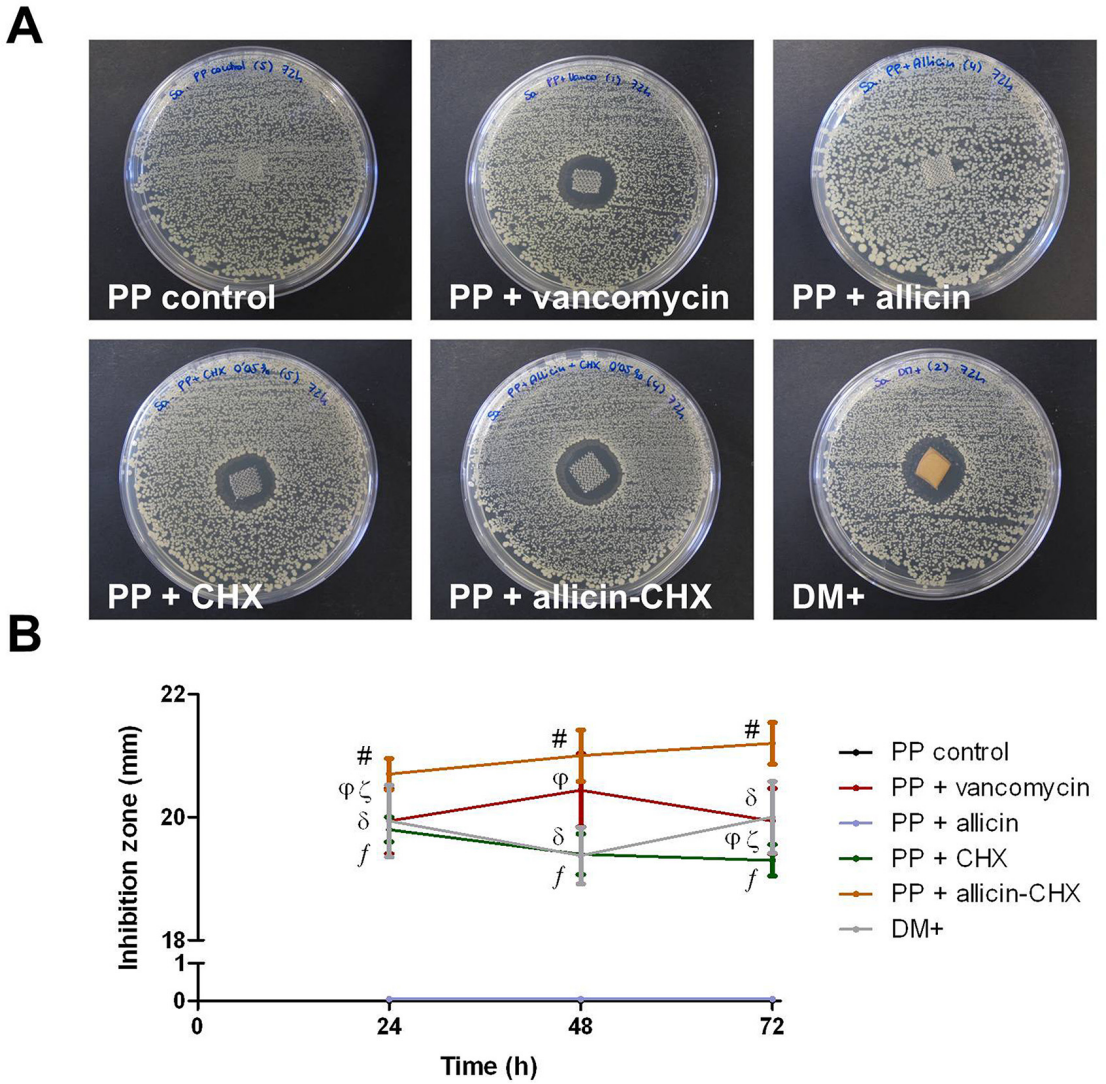
Zones of inhibition created by the pretreated mesh fragments. (A) The images are representative for each study group at 72 h post-contamination with *Sa*. Note the inefficacy of the meshes impregnated with both saline and allicin solutions. (B) Mean diameter of the inhibition zones (mm) created by the pretreated meshes at 24 h, 48 h and 72 h post-contamination. The results are expressed as the mean ± SEM for 5 samples inoculated with *Sa* at each time point. #: PP + allicin-CHX vs PP control, PP + allicin at 24/48/72 h (p<0.01); vs PP + CHX at 24/48/72 h (p<0.05). ƒ: PP + CHX vs PP control, PP + allicin at 24/48/72 h (p<0.01). φ: PP + vancomycin vs PP control at 24/48/72 h (p<0.01); vs PP + allicin at 24/48/72 h (p<0.05). δ: DM+ vs PP control, PP + allicin at 24/48/72 h (p<0.01). ζ: PP + vancomycin vs PP + allicin-CHX 24 h (p<0.05); vs CHX at 72 h (p<0.05).

The PP fragments soaked in saline did not exhibit antibacterial effect against *Sa* (Fig 3A). Similarly, allicin-soaked meshes were not able to create inhibition halos at any of the time-points analyzed. Together with the DM+, all the PP fragments treated with vancomycin, CHX and allicin-CHX exhibited stable inhibition zones between 24 h and 72 h post-contamination, with allicin-CHX producing the widest halos. Due to the lack of inhibition, both the control and the allicin-treated mesh fragments showed statistically relevant differences compared to the rest of the groups at all of the time-points (p<0.05) (Fig 3B). There were no differences in the diameter of the halos among the groups with proven antibacterial effectiveness, with the only exception being the vancomycin-soaked fragments, which showed significantly wider inhibition zones than allicin-CHX at 24 h (p<0.05) and narrower than CHX at 72 h (p<0.05). Table 3 contains the mean data, SEM and percentiles of the inhibition halos produced by the different study groups.

**Table 3:** Mean diameter, SEM, and percentiles of the inhibition zones created by the soaked meshes.

Mean diameter, SEM and percentiles of the inhibition halos (mm)				
Soaked mesh	Data	24 h	48 h	72 h
**PP control**	Mean	No inhibition	No inhibition	No inhibition
	SEM	0	0	0
	Minimum	0	0	0
	25%	0	0	0
	Median	0	0	0
	75%	0	0	0
	Maximum	0	0	0
**PP + vancomycin**	Mean	18.96	20.30	20.50
	SEM	0.45	0.90	0.35
	Minimum	18.00	18.00	19.50
	25%	18.13	18.25	19.75
	Median	19.00	20.50	20.50
	75%	19.78	22.25	21.25
	Maximum	20.56	22.50	21.50
**PP + allicin**	Mean	No inhibition	No inhibition	No inhibition
	SEM	0	0	0
	Minimum	0	0	0
	25%	0	0	0
	Median	0	0	0
	75%	0	0	0
	Maximum	0	0	0
**PP + CHX**	Mean	19.80	19.40	19.30
	SEM	0.20	0.33	0.26
	Minimum	19.50	18.50	18.50
	25%	19.50	18.75	18.75
	Median	19.50	19.50	19.50
	75%	20.25	20.00	19.75
	Maximum	20.50	20.50	20.00
**PP + allicin-CHX**	Mean	20.70	21.00	21.20
	SEM	0.26	0.42	0.34
	Minimum	20.00	20.00	20.00
	25%	20.25	20.25	20.50
	Median	20.50	21.00	21.50
	75%	21.25	21.75	21.75
	Maximum	21.50	22.50	22.00
**DM+**	Mean	19.70	19.30	20.60
	SEM	0.96	0.75	0.53
	Minimum	17.00	17.50	19.00
	25%	17.75	18.00	19.50
	Median	19.50	19.00	20.50
	75%	21.75	20.75	21.75
	Maximum	22.50	22.00	22.00

Data are collected from 5 samples per study group per time point.

**Fig 4:**
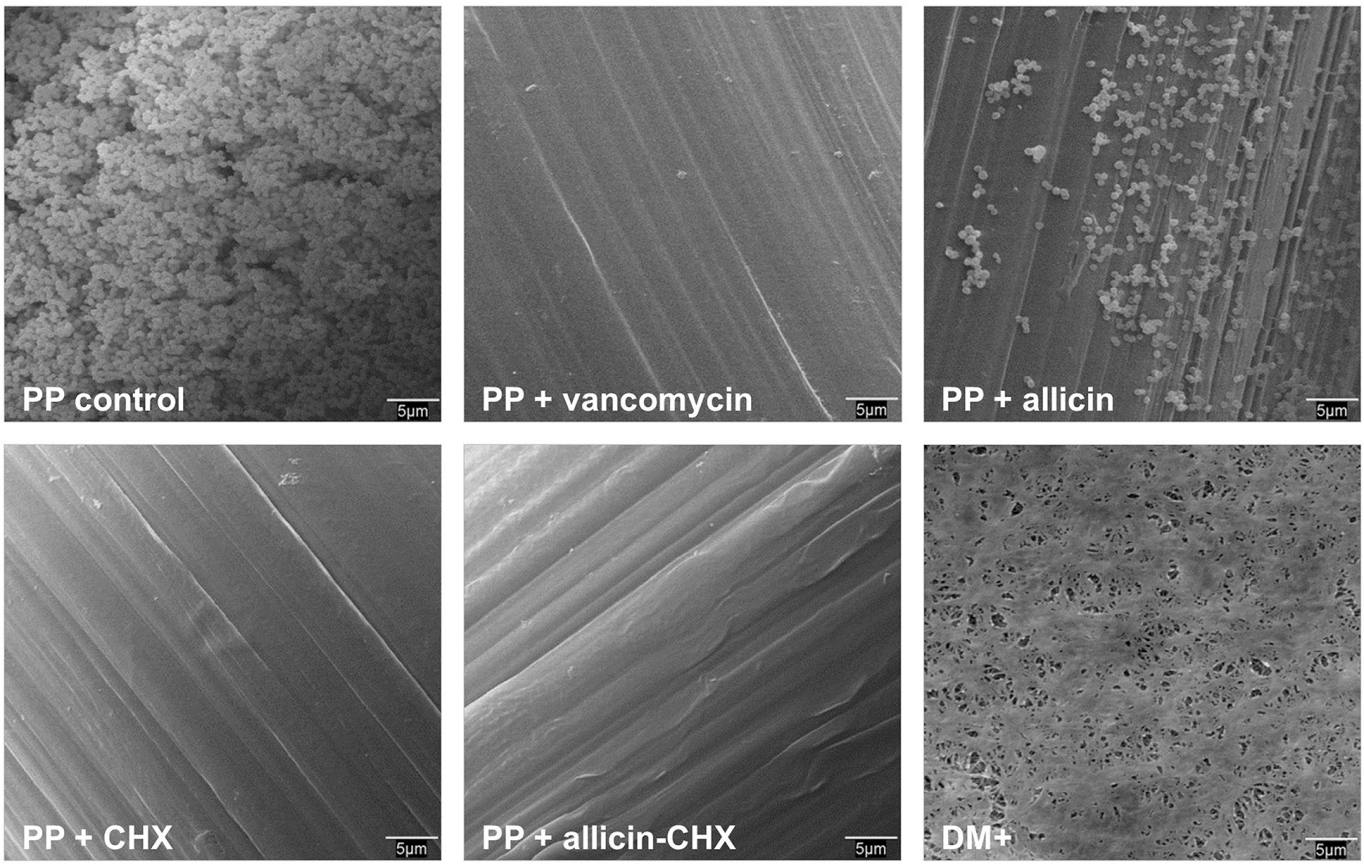
Scanning electron microscopy of the pretreated mesh fragments. The micrographs are representative for each study group at 72 h post-contamination with *Sa* (original magnification 2,000x). The only meshes that show bacteria attached to the material surface were those soaked in saline or allicin. Note that the allicin pretreatment significantly reduced the bacterial load compared to the control meshes.

In accordance with these data, the results from the sonication of the soaked meshes (Table 4) revealed that all the PP fragments belonging to the groups treated with vancomycin, CHX and allicin-CHX, as well as the DM+, had no bacteria attached to the material surface at any of the time-points evaluated. On the contrary, all the samples soaked with saline or allicin showed important bacterial yields after 24 h, 48 h and 72 h post-contamination (p<0.01). Despite this contamination, the bacterial recovery from the surface of the allicin-treated meshes ranged from 2 to 3 logs lower than the control samples (p<0.01). The scanning electron microscopy observations corroborated these findings (Fig 4), confirming that the bacteria were only able to colonize the surface of the control and allicin-treated meshes.

**Table 4:** Mean bacterial loads recovered by sonication of the *Sa*-contaminated meshes.

Bacterial load from the surface of control and allicin coated meshes (CFU/mesh)				
Soaked mesh	Data	24 h	48 h	72 h
**PP control**	Mean	3.22 x 10^10^	4.45 x 10^10^	3.15 x 10^9^
	SEM	3.07 x 10^10^	2.50 x 10^10^	1.24 x 10^9^
	Minimum	8.20 x 10^8^	7.60 x 10^8^	8.40 x 10^8^
	25%	1.23 x 10^9^	1.09 x 10^9^	8.75 x 10^8^
	Median	1.77 x 10^9^	1.25 x 10^10^	1.70 x 10^9^
	75%	7.84 x 10^10^	1.04 x 10^11^	6.15 x 10^9^
	Maximum	1.55 x 10^11^	1.22 x 10^11^	6.60 x 10^9^
**PP + allicin**	Mean	2.93 x 10^7^	6.10 x 10^7^	2.19 x 10^7^
	SEM	1.70 x 10^7^	2.86 x 10^7^	1.02 x 10^7^
	Minimum	1.17 x 10^6^	4.60 x 10^6^	4.60 x 10^6^
	25%	2.00 x 10^6^	1.07 x 10^7^	9.00 x 10^6^
	Median	2.11 x 10^7^	3.26 x 10^7^	1.36 x 10^7^
	75%	608 x 10^7^	1.26 x 10^8^	3.90 x 10^7^
	Maximum	9.40 x 10^7^	1.58 x 10^8^	6.20 x 10^7^

Data are collected from 5 samples per study group per time point. The samples from the vancomycin, CHX, allicin-CHX and DM+ groups exhibited no bacterial load on the surface of the meshes.

### Cell viability

**Fig 5:**
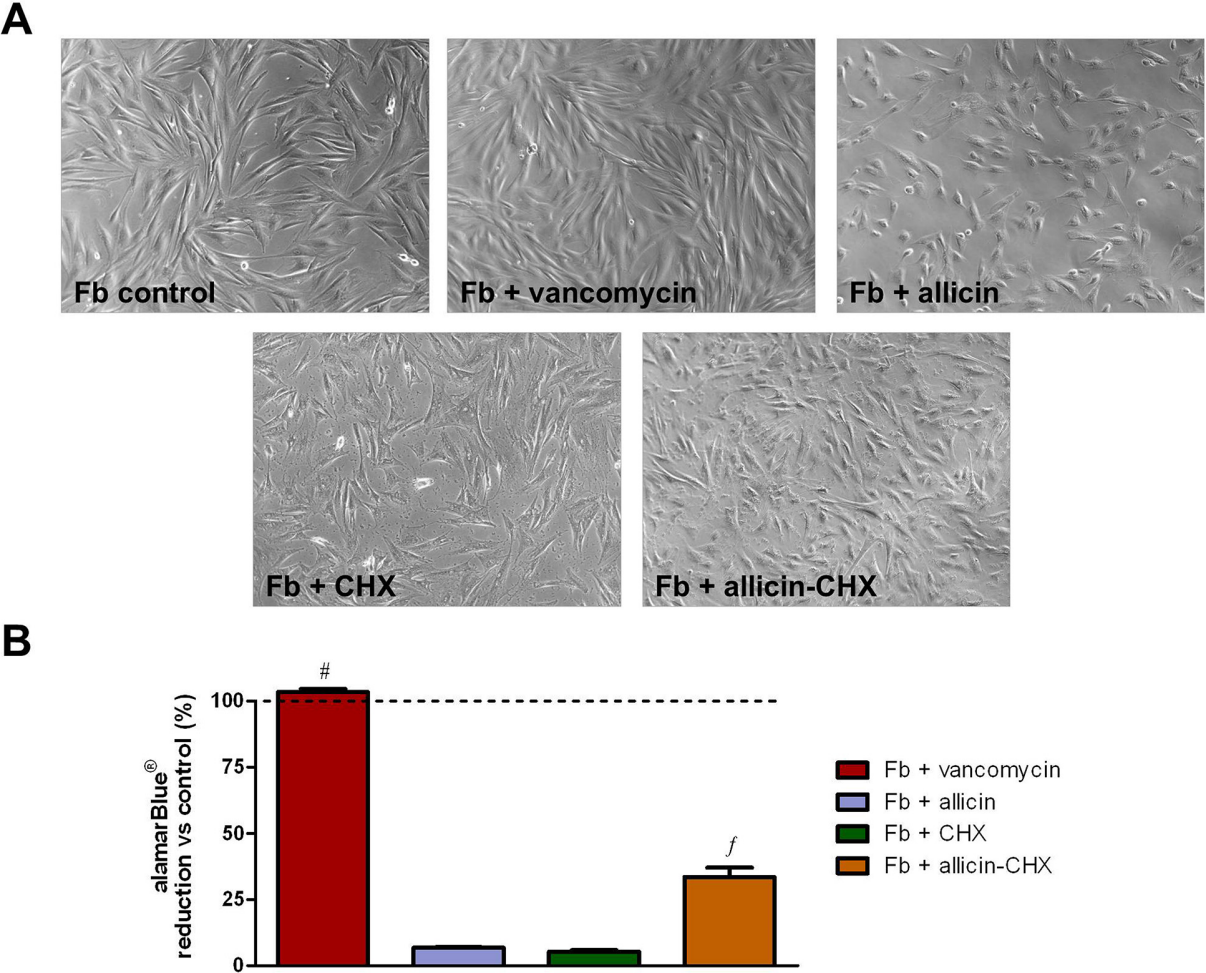
Cell viability. (A) Micrographs of the cultured fibroblasts after a 24 h exposure to the different antibacterial treatments (original magnification 100x). (B) Mean percentage of the alamarBlue reduction from cells treated with the antibacterial solutions vs non-treated cells after 24 h of culture. The results are expressed as the mean ± SEM for 3 independent assays performed in quadruplicate. #: Fb + vancomycin vs Fb + allicin, Fb + CHX, Fb + allicin-CHX (p<0.001). ƒ: Fb + allicin-CHX vs Fb + allicin, Fb + CHX (p<0.001).

Approximately 15–20 days after their isolation, fibroblasts migrated from the explants and started growing on the flask surface. The cells displayed an elongated shape and multilayered growth (Fig 5A). When the cells were exposed to media containing antibacterial treatments, no evidence of morphological changes were observed in the vancomycin-treated group after 24 h of culture. However, the shape of the fibroblasts treated with allicin turned more polygonal or even rounded, exhibiting a discrete cell detachment from the substrate. The fibroblasts treated with both CHX and allicin-CHX turned slightly stellate-shaped and the wells showed small amounts of solid precipitate due to the interaction between the CHX and the salts in the medium. The results from the alamarBlue assays (Fig 5B and Table 5) demonstrated no cytotoxic effect of vancomycin, with mean percentage alamarBlue reduction vs control cells of 103.40 ± 1.09%. Contrary to this, both allicin and CHX treatments provoked an acute decrease in the metabolic activity compared to the control (6.88 ± 0.26% and 5.34 ± 0.57%, respectively; p<0.001). Surprisingly, the fibroblasts treated with the combination of allicin-CHX, although exhibiting a decrease in metabolic activity compared to the control and vancomycin-treated cells (p<0.001), showed a significantly higher metabolic activity that the groups treated with either allicin or CHX alone (33.58 ± 3.44%; p<0.001).

**Table 5:** Mean data, SEM and percentiles of the alamarBlue reduction from cells treated with the antibacterial solutions vs non-treated cells after 24 h of culture.

Mean data and percentiles of the alamarBlue reduction (%)		
Treated cells	Data	24 h
**Fb + vancomycin**	Mean	103.40
	SEM	1.09
	Minimum	95.10
	25%	101.00
	Median	103.70
	75%	105.30
	Maximum	109.20
**Fb + allicin**	Mean	6.88
	SEM	0.26
	Minimum	5.50
	25%	5.98
	Median	7.00
	75%	7.60
	Maximum	8.00
**Fb + CHX**	Mean	5.34
	SEM	0.57
	Minimum	2.60
	25%	3.13
	Median	5.60
	75%	7.33
	Maximum	7.70
**Fb + allicin-CHX**	Mean	33.58
	SEM	3.44
	Minimum	16.50
	25%	18.33
	Median	40.60
	75%	40.98
	Maximum	46.80

Data are collected from 3 experiments carried out in quadruplicate.

## Discussion

Hernia mesh infection represents an important complication in the clinical setting not only due to the associated economic costs, but also because these surgical procedures currently develop frequently in patients with other concomitant pathologies (i.e., diabetes, vascular diseases). Together with these underlying issues, the activity of the patient’s immune system is obviously another risk factor to be considered [11].

Bacterial adhesion to the material surface during the first stages of mesh colonization is a key process that triggers the development of implant infection. If this happens, the bacteria can develop biofilms on the mesh or host tissue surfaces, compromising the performance of the implant. Inside the biofilm, bacteria are protected by an exopolysaccharide matrix that increases their resistance to the action of drugs (i.e., antibiotics) and host immune cells [10]. Furthermore, some bacteria have the ability to detach from the biofilm, migrate and colonize other regions [20], forming novel biofilms and thus increasing the virulence of the infection. Finally, and due to the low effectiveness of the systemic antibiotic treatments in the setting of biofilm infections, in many cases the only viable alternative is the partial or total removal of the contaminated implant [21,22], with the consequent economic cost and increase in the patient’s morbidity [23].

This situation highlights the necessity of designing medical devices charged with effective antimicrobial properties to control bacterial adhesion to the material surface and thus reduce the risk of biofilm formation [16,24]. In this regard, coating the mesh with antimicrobial compounds could represent a suitable strategy [25]. In the clinical setting and immediately prior to implantation, the hernia mesh materials are often soaked or dipped in antibiotics such as gentamicin [25,26] or vancomycin [27]. However, despite this being a common practice, the effectiveness of the mesh impregnation to control bacterial infections is not yet conclusive [27,28].

In the present work, we have evaluated the performance of several antibacterial treatments, one antibiotic (vancomycin) and two antiseptics (allicin, CHX). Specifically, allicin has not been previously used to impregnate hernia mesh materials. In this *in vitro* model, we have chosen a *Sa* strain at a high dose (10^6^ CFU) as the contaminating microorganism to provoke an acute contamination not only in the mesh surface but also on the agar plates utilized as a substrate. This dose is notably higher than the one which can lead to a surgical site infection because a bacterial load of 10^2^ CFU is enough to trigger an infection in patients undergoing prosthetic mesh repair [29]. Therefore, this experimental design allowed us to test the effectiveness of the treatments in a severe case of infection.

When we treated a contaminated substrate in the absence of mesh, both vancomycin and CHX were effective at the concentrations tested, provoking wide and stable inhibition halos. Curiously, the allicin solution lost its properties after 24 h, allowing the bacterial colonization of the inhibition areas previously formed on the agar plates. The best activity was observed with the allicin-CHX combination because the antibacterial effect of this mixed solution against *Sa* was significantly more powerful than the one provoked by vancomycin or CHX. When we utilized these solutions to soak the PP fragments and exposed them to a contaminated substrate, we could observe once again a higher performance of the fragments soaked with allicin-CHX compared to the vancomycin and CHX-soaked meshes. Additionally, the samples treated with this combination of antiseptics developed inhibition zones slightly wider than those observed with DM+, up to now known for being the only FDA-approved hernia mesh material exerting antibacterial effects [15].

Our results suggest a complementary relationship between allicin and CHX. Allicin is a natural antiseptic that has been shown to have a wide antimicrobial spectrum against gram-positive bacteria, gram-negative bacteria and fungi [17]. Nevertheless, its effectiveness is highly dependent on the variety of garlic and the extraction and purification processes [30]; thus, it is necessary to control the origin of the compound. The allicin solution that we utilized was previously tested *in vitro* against methicillin-resistant *Staphylococcus aureus* (MRSA) [31] and Lancefield group B streptococci [32] with promising results.

To our knowledge, this is the first time that this natural antiseptic is being used in combination with CHX. It has been reported that the antibacterial activity of allicin can be enhanced both *in vitro* and *in vivo* when it is applied together with other agents, whether they are antibiotics or not. Recent studies have demonstrated that a combination of allicin with vancomycin is more effective than both agents alone in an experimental model of prosthetic joint infection caused by *Se* [16]. Similarly, the combined use of allicin and silver nanoparticles exerts a more intense effect in the disinfection of MRSA-contaminated wounds [33]. These data agree with the hypothesis that the combination of two or more antibacterial compounds could enhance the performance of the treatment because the antibacterial spectrum is increased and the risk of developing resistances is reduced [34].

When selecting an antimicrobial agent to be utilized together with a medical device, it is important to find a balance between the effectiveness and the toxicity of the treatment. At the concentration tested, vancomycin demonstrated good antibacterial effects and no cell toxicity. Despite this effectiveness, vancomycin is an antibiotic and its extended use can lead to the rise of resistant strains [35]; thus, vancomycin application with medical devices should be limited. Allicin not only showed high cytotoxicity but also exhibited a bacteriostatic behavior, which could imply the need for periodic administration to retain its activity. The antibacterial and cytotoxic effects of CHX, even at the low concentrations utilized in this work, are well reported both *in vitro* [36] and *in vivo* [37]. However, our findings indicate that the cell toxicity of CHX is notably reduced when it is combined with allicin, without compromising its strong antibacterial effect against *Sa*. It has been recently demonstrated that cyclic oligosaccharides such as cyclodextrins can interact with CHX, increasing the antibacterial effect against several microorganisms and simultaneously decreasing the toxicity towards eukaryotic cells [38]. While the mechanism of interaction between these two compounds is not yet well understood, in the light of these findings we could hypothesize that allicin can act as a modulator agent of CHX activity, reducing its toxicity to the fibroblasts and simultaneously improving its antibacterial effect against *Sa*.

The positive effects of the mesh soaking we have reported could be enhanced by coating the material surface with bioactive polymers capable of releasing antimicrobial substances in a local and controlled fashion, as we previously demonstrated with an *in vitro* and *in vivo* model of acute infection caused by *Sa* and *Se*, using polymer-coated lightweight PP meshes with a controlled release of vancomycin [39]. The use of bioactive meshes would be advantageous because these coatings can supply a continuous release of the antibacterial compound during the first 24–72 h post-implantation. This approach would avoid the rise of antibiotic resistances because lower drug concentrations would be needed and would simultaneously reduce the local toxicity [40]. Additionally, these bioactive coatings can be designed with antiadhesive properties [41] and can be applied on different medical devices, regardless of their structure, shape or dimensions [39].

To conclude, our findings demonstrate that soaking mesh with antiseptics such as CHX and the combination allicin-CHX produces similar or stronger antibacterial effects than those provoked by the antibiotic vancomycin. Although these findings are very promising, this is an *in vitro* study and is therefore not easy to extrapolate to the clinical setting. It is well known that, after implantation, the biomaterial is covered by a thin aqueous layer containing proteins (such as fibronectin, fibrinogen or collagen, among others). The presence of this conditioning film can stimulate the adhesion to the mesh surface and adjacent tissues of both bacteria and host cells, in a process known as “race for the surface” [42]. Theoretically, if the mesh is soaked in antibacterial solutions prior to the implantation, the treatment would prevent the interaction of the bacteria with the proteins of the conditioning film, and thus the treatment would reduce the risk of developing surgical site infections. By controlling the bacterial adhesion, the mesh soaking would facilitate the arrival of host cells to the wound area, cooperating in the healing process. Taking into account that we have developed an *in vitro* model, our results cannot predict how these soaked meshes will behave under *in vivo* conditions. Accordingly, we consider that it is necessary to develop *in vivo* assays to provide more data regarding the potential application of CHX, alone or in combination with allicin, in the coating of prosthetic materials for hernia repair. Additionally, these assays will provide important details related to the role of the antimicrobial mesh coating in the processes of mesh integration and tissue regeneration.
